# Electrochemical Sensors and Biosensors Represent Very Promising Tools in Pharmaceutical Sciences

**Published:** 2015

**Authors:** Fatemeh Ghorbani-Bidkorbeh

**Affiliations:** *Department of Pharmaceutics, School of Pharmacy, Shahid Beheshti University of Medical Sciences, Tehran, Iran.*


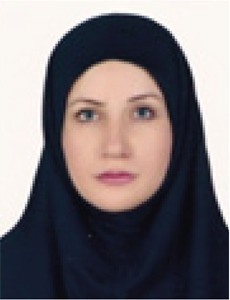


The explosive development of knowledge, technology and human society has resulted in urgently needs of more sensitive, specific, reliable, efficient, continuous, cost-effective, faster and conveniently analytical methods with miniaturized and portable devices to monitor key parameters. Electrochemical sensors and biosensors have been one of the most attractive areas of research and have found a vast range of pharmaceutical, biomedical, clinical, industrial, environmental and agricultural applications to meet these requirements and offer a better quality of drugs, food, environment, and more general, a better quality of life.

Electrochemical sensors hold a leading position among chemical sensors which consist of a signal transducer covered by a recognition element, and can be utilized for fast, simple and direct analysis in an even turbid sample matrix. Furthermore, biochemical molecular recognition properties such as antibody-antigen binding and enzymatic reactions are employed in biosensors for selective analysis. In electrochemical sensors, an electrode is used to transduce a biological or chemical signal into an electrical signal by electroanalytical techniques such as potentiometry, conductometry and amperometry (voltammetry), which are based on potential, conductivity and current measurement, respectively. Up to the 1960s, pH glass electrode as a chemical sensor and the amperometric glucose enzyme electrode as the modern concept of biosensors were the only sensors introduced. However, with the start of the 21st century, the evolution of sophisticated sensing devices has been observed and furthermore, research into electrochemical sensor design is developing in new exciting directions and applications. As a dramatically multidisciplinary field of science from electrochemistry, biochemistry, biophysics, cell biology, molecular biology, biomedicine, analytical chemistry, biomedical engineering, nanotechnology, biotechnology, and cancer research, future progress would require extensive efforts for fulfilling emerging needs from drug and food analysis to early detection of disease biomarkers.

This fascinating field of science is particularly concerned with understanding electrical phenomena in biological systems as can be seen in biological events such as signal transduction, metabolism and energy conversion with *in-vivo *changes of current or potential. Therefore, electrochemical analysis can reflect the oxidation or reduction (electron transfer) of ions, oligonucleotides and enzymes. These phenomena have been documented by Nobel prizes in medicine in 1936 for the chemical basis prove of neurotransmitter release, in 1963 for the sodium-potassium ion pump model of nerve impulses, in 1970 for mechanisms of humoral transmission in nerve cells, in 1978 for chemiosmosis of the electrochemical membrane gradient that drives ATP synthesis; in 1991 for understanding of the function of single-ion channels; in 1997 in chemistry for discovery of the ion-transporting enzyme Na+, K+-ATPase, and continuing in 2000 in medicine, for signal transduction in the nervous system. Furthermore, the electroanalysis of neurotransmitters in living brain cells is approaching a state of maturity. In addition, charge transport along the DNA double helix has been demonstrated and the development of electroanalytical polynucleotide hybridization sensors using nucleic acids recognition layers promises to be a foundation of future DNA biosensing devices. Electroanalysis of proteins especially redox enzymes, cells and functional components inside cells has attracted particular interests due to their essential role and is of significant importance in early detection of diseases. Over the past two decades, electrochemical behaviour of drugs such as ascorbic acid, phenothiazines, benzodiazepines, paracetamol, theophylline, tramadol, sumatriptan, gabapentin, lamotrigine, etc. has been investigated and a great trend in pharmaceutical research can be seen for developing electrochemical sensors. In addition, intelligent drug delivery systems or self-contained implantable sensors for the rapid detection of biomarker and the release of therapeutic agents on demand can be designed utilizing these sensors.

Nevertheless, since the electron transfer rates between biomolecules and electrode surfaces are usually prohibitively slow, bioelectroanalysis is often difficult to be performed. In this regard, electrochemistry is gradually moving beyond some disadvantages with the rapid development of electrode modifications applying conducting polymers, molecular recognition elements like molecular imprinted polymers and aptamers, nanomaterials and nanoconstructions such as gold nanoparticles, carbon nanoparticles, carbon nanotubes, graphene nanosheets, magnetic nanoparticles, etc.

Therefore, electrochemical sensors and biosensors are excellent candidates for immunoassays and determination of pharmaceuticals, DNA, amino acids, peptides, proteins, and cells *in-vivo *and *in-vitro *determination of in extremely small and complex biological samples due to their high sensitivity and selectivity coupled to their compatibility with nanotechnology and modern miniaturization/ microfabrication technologies such as lab-on-a-chip, low cost, minimal power requirements, and independence of sample turbidity Pharmaceutical scientists can take advantages of this great opportunity in pharmacology, toxicology and pharmaceutical sciences.

*Fatemeh Ghorbani-Bidkorbeh* *is currently working as an assistant professor at the Department of Pharmaceutics, School of Pharmacy, Shahid Beheshti University of Medical Sciences, Tehran, Iran. She could be reached at the following e-mail address:* *f.ghorbani@sbmu.ac.ir*

